# Multi-kernel feature extraction with dynamic fusion and downsampled residual feature embedding for predicting rice RNA *N*^6^-methyladenine sites

**DOI:** 10.1093/bib/bbae647

**Published:** 2024-12-14

**Authors:** Mengya Liu, Zhan-Li Sun, Zhigang Zeng, Kin-Man Lam

**Affiliations:** School of Computer Science and Technology, Anhui University, Hefei 230601, China; School of Electrical Engineering and Automation, Anhui University, Hefei 230601, China; School of Artificial Intelligence and Automation, Huazhong University of Science and Technology, Wuhan 430074, China; Department of Electronic and Information Engineering, The Hong Kong Polytechnic University, Hong Kong, China

**Keywords:** RNA N^6^-methyladenine, multi-kernel feature, global–local dynamic fusion, downsampling residual embedding, rice genome

## Abstract

RNA *N*$^{6}$-methyladenosine (m$^{6}$A) is a critical epigenetic modification closely related to rice growth, development, and stress response. m$^{6}$A accurate identification, directly related to precision rice breeding and improvement, is fundamental to revealing phenotype regulatory and molecular mechanisms. Faced on rice m$^{6}$A variable-length sequence, to input into the model, the maximum length padding and label encoding usually adapt to obtain the max-length padded sequence for prediction. Although this can retain complete sequence information, resulting in sparse information and invalid padding, reducing feature extraction accuracy. Simultaneously, existing rice-specific m$^{6}$A prediction methods are still at an early stage. To address these issues, we develop a new end-to-end deep learning framework, MFDm$^{6}$ARice, for predicting rice m$^{6}$A sites. In particular, to alleviate sparseness, we construct a multi-kernel feature fusion module to mine essential information in max-length padded sequences by multi-kernel feature extraction function and effectively transfer information through global–local dynamic fusion function. Concurrently, considering the complexity and computational efficiency of high-dimensional features caused by invalid padding, we design a downsampling residual feature embedding module to optimize feature space compression and achieve accurate feature expression and efficient computational performance. Experiments show that MFDm$^{6}$ARice outperforms comparison methods in cross-validation, same- and cross-species independent test sets, demonstrating good robustness and generalization. The application on maize m$^{6}$A indicates the MFDm$^{6}$ARice’s scalability. Further investigations have shown that combining different kernel features, focusing on global channel-local spatial, and employing reasonable downsampling and residual connections can improve feature representation and extraction, ensure effective information transfer, and significantly enhance model performance.

## Introduction

There are over 200 post-transcriptional epigenetic modifications of RNA in eukaryotes, such as *N*$^{6}$-methyladenosine (m$^{6}$A), *N*$^{1}$-methyladenosine (m$^{1}$A), 5-methylcytidine (m$^{5}$C), 1-methylguanosine (m$^{1}$G), pseudouridine ($\psi $) [1]. The most prevalent internal modification is m$^{6}$A, which occurs widely in mRNA, miRNA, long non-coding RNA, tRNA, and rRNA [2]. Methyltransferase complexes (writers), demethylases (erasers), and m$^{6}$A-binding proteins (readers) are the primary components of the m$^{6}$A modification system. Writers and erasers add and remove the methyl group (–CH$_{3}$) to the amino group (–NH$_{2}$) at the sixth position of adenosines in the RNA, respectively. Readers recognize the m$^{6}$A site and play specific regulatory roles [3]. In *Oryza sativa* (rice), m$^{6}$A is involved in growth, development [4–6], and response to biotic [7–10] and abiotic stresses [11–14]. For example, m$^{6}$A regulates the early degeneration of rice microspores at the vacuolar pollen stage [4]. Dynamic regulation of m$^{6}$A occurs during rice-plant virus interactions [7]. Cheng *et al*. found that rice m$^{6}$A may be associated with cadmium stress-induced aberrant root development [11].

MeRIP-seq (m$^{6}$A-seq) allows the detection of m$^{6}$A sites in plants but has a resolution of 100–200 nucleotides, which is too coarse for precise m$^{6}$A editing detection [15, 16]. Several improvements with single-base resolution have been developed, including PA-m$^{6}$A-seq [17], miCLIP [18], and m$^{6}$A-CLIP-seq [19]. However, these techniques are limited by the small sample sizes of high-RNA-metabolism tissues, making biological replication and accurate detection difficult [19]. The DART-seq method [20] requires only 10 ng of RNA, much less than MeRIP-seq. Advances in third-generation sequencing technologies, such as single-molecule real-time sequencing [21] and nanopore sequencing [22], have also enabled better detection of m$^{6}$A sites. In 2020, Parker *et al*. successfully used nanopore direct RNA sequencing to map m$^{6}$A sites in *Arabidopsis* [23]. This technique is well-suited for small samples and can significantly accelerate m$^{6}$A research in plants, aiding the mapping of single-base resolution modifications and editing.

Although bio-experimental m$^{6}$A site detection technology is continuously being improved and refined, it still requires substantial human, material, and financial resources. Therefore, there is an urgent need to develop corresponding computational methods. Currently, some m$^{6}$A site prediction methods have been proposed for many species, especially *Homo sapiens* [24–28]. In plant research, several computational methods have also been developed to predict m$^{6}$A sites across different species. For instance, SMEP [29] is a method designed for rice and *Zea mays* (maize), while m6A-Maize [30] focuses exclusively on maize. PEA-m6A [31] has been applied to various economically important plants, including rice, maize, and *Triticum aestivum L.* (wheat), showing its potential applicability across multiple crops. For *Arabidopsis*, models like RFAthM6A [32] and M6AMRFS [33] have also been proposed.

Despite rice’s economic importance, few methods exist for predicting m$^{6}$A sites in rice. In 2021, Wang and colleagues introduced SMEP [29], the first computational method for rice m$^{6}$A site prediction, after collecting and processing the first rice m$^{6}$A dataset. SMEP used padding and label encoding to handle variable-length m$^{6}$A sequences and applied convolutional layers to extract high-level features, followed by a multilayer perceptron for final prediction. While SMEP laid the foundation for rice m$^{6}$A site prediction, its use of max-length padding leads to sparse features, affecting extraction, and the multiple convolutional layers focus mainly on local information, missing broader context. Recently, Song *et al*. proposed PEA-m6A [31], an ensemble learning method based on gradient-boosted decision trees, which integrates statistical and deep learning features to improve feature representation and achieve strong performance. However, PEA-m6A is not an end-to-end model and struggles with unstructured data. Additionally, simply stacking features from different sources has limitations for enhancing feature representation.

Considering the advantage of max-length padded sequences that retain complete information, to exploit the information they encode and overcome existing limitations fully, we develop an end-to-end **Rice m$^{6}$A** site prediction learning framework called **MFDm$^{6}$ARice**. This framework combines **M**ulti-kernel feature extraction, dynamic **F**usion of global and local features, and **D**ownsampled residual embedding technology. Since variable-length sequences contain valuable information, we use label encoding and padding to construct max-length padded sequences to facilitate feature learning. However, this padding can lead to sparse and redundant features, reducing the model’s ability to capture meaningful sequence-level information. To this end, we develop a multi-kernel feature fusion (MKFF) module that extracts key features across multiple receptive fields to reduce sparsity. These features are then effectively fused and transferred by combining global and local features. To improve computational efficiency, we introduce a downsampling residual feature embedding (DRFE) module, which compresses features efficiently and enhances performance. Experiments show that MFDm$^{6}$ARice surpasses state-of-the-art methods in performance, robustness, and generalization. Its scalability is demonstrated with maize m$^{6}$A data. Importantly, comparative experiments confirm that global–local dynamic fusion (GLDF) of multi-kernel features, appropriate downsampling, and residual connections significantly enhance feature representation and model performance.

## Materials and methods

### Datasets

In this study, one benchmark dataset and two independent test sets are used to evaluate the performance of MFDm$^{6}$ARice. Table 1 tabulates the details of these datasets.

**Table 1:** Details of benchmark dataset and independent test sets

Datasets	Number of positives	Number of negatives	Total number
Benchmark dataset	19 844	39 692	59 536
Same-species independent test set	4963	9923	14 886
Cross-species independent test set	24 532	24 518	49 050

Wang *et al*. [29] proposed the first rice m$^{6}$A dataset, with positive samples extracted from m$^{6}$A-seq peak sequences of *Japonica* Nipponbare seedling leaves, ranging from 20 to 800 nt in length. Negative samples were selected by extracting equal-length sequences without m$^{6}$A sites from the upstream and downstream regions of the positive samples. They used CD-HIT [34] to reduce homology bias and remove redundant sequences. After cleaning and deduplication, 80% of the positive and negative samples were randomly selected as the benchmark dataset for evaluating model performance. The final prediction model was tuned and trained, and its generalizability was tested using independent test sets. The remaining samples were used as a same-species independent test set, and *H. sapiens* m$^{6}$A data from DeepM6ASeq [26] were used as a cross-species independent test set. Unlike the peak sequences in the benchmark and same-species sets, the cross-species test set contained precise methylation sites with 101 nt-long positive and negative samples. Notably, these data had been mapped to the reference genome and provided as the corresponding DNA sequences (A, T, G, and C).

### Architecture of MFDm$^{6}$ARice

**Figure 1:**
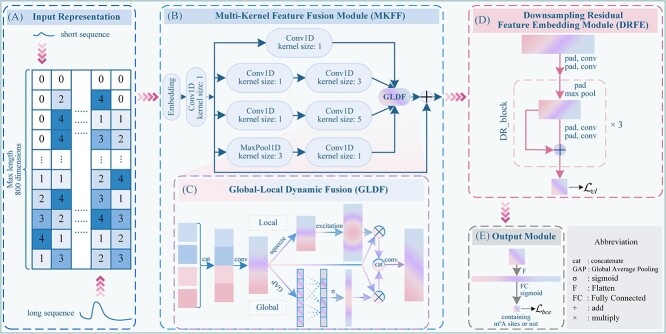
Overall flowchart of MFDm$^{6}$ARice. (A) Input representation of m$^{6}$A sequences. Label encoding is performed on sequences after padding to the maximum length of 800 to obtain max-length padded sequences. (B) After the input representation, it is input into the MKFF module. Its multi-kernel feature extraction function extracts multi-kernel features (the upper part of B), then inputs these features into the (C) GLDF function to obtain multi-kernel dynamic fused features. (D) The dynamic fused features are input into the DRFE module to get the corresponding feature embedding. DR_block represents the downsampling residual block. (E) Finally, the output module predicts whether contained m$^{6}$A sites or not.

Figure 1 shows the architecture of MFDm$^{6}$ARice framework. The framework consists of four main modules: input representation module, MKFF module, DRFE module, and output module.

#### Input representation

To adapt the input to the model, it is first necessary to convert the m$^{6}$A sequence into a numerical representation. The label encoding method is a simple yet effective approach for transforming classified text data into numerical form, assigning each category a unique numerical identifier [35]. Accordingly, we utilize this technique to represent the input m$^{6}$A sequences in this study. In particular, m$^{6}$A sequences are variable-length data that have rich information. To retain complete sequence information and incorporate it into the model, like SMEP, we pad all sequences to the maximum length of 800 to obtain the max-length padded sequences, of which each sequence has five categories. For example, assuming the maximum sequence length is 10, a sequence ‘ATTCG’ consisting of four type bases (A, T, G, and C) pads to ‘PPPPPATTCG’ where ‘P’ denotes padding. The five categories (P, A, T, G, and C) are assigned numbers by label encoding: 0, 1, 2, 3, and 4, respectively. Consequently, this max-length padded sequence can be represented as $LE{\_ }Fea=[0, 0, 0, 0, 0, 1, 2, 2, 4, 3]$, as shown in Fig. 1A. In addition, we present an experimental analysis of padding length in the Supplementary Section S1 and Supplementary Table S1.

#### Multi-Kernel feature fusion module

Although max-length padded sequences can preserve complete sequence-level information, they also introduce lengthy and ineffective information. Such can lead to sparse and redundant features that hinder the model’s ability to capture subsequent meaningful sequence-level information. Therefore, after obtaining the m$^{6}$A feature representation ($LE{\_ }Fea$), we design an MKFF module that combines the multi-kernel feature extraction function and GLDF function to extract and integrate features from different kernels, as shown in Fig. 1B and C.

***Multi-Kernel feature extraction function.*** To help mitigate the sparsity problem associated with max-length padded sequence encoding, we develop the multi-kernel feature extraction function, which extracts crucial features across multiple receptive fields. This function guarantees efficacious feature capture from different scales and regions, providing a more comprehensive feature representation and contextual information of the sequence. Specifically, firstly, the $LE{\_ }Fea$ is passed through the embedding layer ($Embedding$) to obtain embedding vectors. Besides, for embedding vectors, a point-wise convolution (i.e. a kernel size of 1, $Conv_{1}$) is used to transform dimension and feature to obtain $O_{embed}$. As follows: 


(1) \begin{align*} O_{embed}&=Conv_{1}(Embedding(LE{\_}Fea))\end{align*}

Then, four parallel paths are operated on $O_{embed}$ to extract information at different kernel receptive fields: 


(2) \begin{align*} k1&=Conv_{1}(O_{embed})\qquad\qquad \end{align*}

(3) \begin{align*} k2&=Conv_{3}(Conv_{1}(O_{embed}))\ \ \end{align*}

(4) \begin{align*} k3&=Conv_{5}(Conv_{1}(O_{embed}))\ \ \end{align*}

(5) \begin{align*} k4&=Conv_{1}(MaxPool_{3}(O_{embed})) \end{align*}

where (2), (3), and (4) use convolution layers with kernel sizes of 1, 3, and 5 ($Conv_{1}, Conv_{3}, Conv_{5}$), respectively, to extract information of different spatial dimensions. The rationale for choosing these kernel sizes is provided in Supplementary Section S2. In addition, we also perform experimental analysis on other kernel sizes, such as 7 and 9, see Supplementary Section S2 and Table S2. Equations (3) and (4) initially perform $Conv_{1}$ on the input to reduce the number of channels, thereby decreasing the number of parameters and the complexity of the model. Equation (5) uses a max pooling layer with a kernel size of 3 ($MaxPool_{3}$) and then employs $Conv_{1}$ to change the number of channels. Appropriate padding is applied to these paths to maintain consistent input and output heights and widths. $k1$, $k2$, $k3$, and $k4$ are the multi-kernel features obtained.

***Global-local dynamic fusion function.*** Having obtained multi-kernel features, if merely concatenating them is simple and practical, it does not consider the differences between the features at various kernels. Therefore, it is pivotal to integrate the multi-kernel features and efficiently transfer them. For this reason, we propose a GLDF function as an alternative to the simple concatenation process, ensuring the preservation of broad contextual information and fine-grained details.

This function contains two main parts: global channel dynamic fusion and local spatial dynamic fusion, as shown in Fig. 1. The lower part is global channel dynamic fusion, the upper part is local spatial dynamic fusion. Details are as follows.

(i) Feature concatenation

The first step is to concatenate the extracted multi-kernel features. After, $Conv_{1}$ is applied to perform point-wise channel information interaction and context aggregation at each spatial position. 


(6) \begin{align*} MK{\_}Fea=Conv_{1}(cat(k1, k2, k3, k4))\end{align*}

(ii) Global channel dynamic fusion

In global channel dynamic fusion, channel aggregation features are first generated by compressing the global spatial information to the channel using global average pooling $AvgPool$. The global channel attention weights $Global_{weight}$ are then dynamically generated by convolution with kernel size $Conv_{k_{adp}}$ and the $Sigmoid$ nonlinearity function. 


(7) \begin{align*} Global_{weight} = Sigmoid(Conv_{k_{adp}}(AvgPool(MK{\_}Fea)))\end{align*}

where $k_{adp}$ represents the adaptive convolution kernel size, which is used to flexibly capture the dependencies between different channels [36]. Given the channel dimension $C$, it is calculated as follows: 


(8) \begin{align*} k_{adp}=\left|\frac{\log_{2}(C)}{\gamma} + \frac{b}{\gamma} \right|{{}}_{odd}\end{align*}

where $\left | \right |{}_{odd}$ means rounding down the absolute value to the nearest odd number. $\gamma $ and $b$ are two hyperparameters. As with ECA [36], they are set to 2 and 1, respectively.

Therefore, the global dynamic fusion features can be obtained by the following equation: 


(9) \begin{align*} Global{\_}Fea&=MK{\_}Fea \otimes Global_{weight}\end{align*}

where $\otimes $ is the element-wise multiplication.

(iii) Local spatial dynamic fusion

For local spatial dynamic fusion, the spatial attention weights $Local_{weight}$ are calculated through a bottleneck structure as follows: 


(10) \begin{align*} squeeze&=Conv_{k_{adp}}^{squeeze}(MK{\_}Fea)\qquad\qquad \end{align*}

(11) \begin{align*} Local_{weight}&=Softmax(Conv_{k_{adp}}^{excitation}(squeeze))\end{align*}

Given the input channel dimension $C$, the output channel of $Conv_{k_{adp}}^{squeeze}$ and the input channel of $Conv_{k_{adp}}^{excitation}$ are $C//r$, and the output channel of $Conv_{k_{adp}}^{excitation}$ is $C$. Here, $r$ is the reduction ratio [37], a key hyperparameter. $Softmax$ is the softmax function. Specifically, $Conv_{k_{adp}}^{squeeze}$ is applied to the $MK{\_ }Fea$, compressing the spatial features into a compact representation while retaining essential information. This squeezing operation reduces capacity and computational cost. Subsequently, $Conv_{k_{adp}}^{excitation}$ and a non-linear transformation softmax function are employed to activate the information aggregated during the squeeze operation. These operations allow dynamic weighting of spatial features, facilitating the capture of dependencies between spatial features and enhancing critical features.

After obtaining the local spatial attention weights, $Local_{weight}$, the local dynamic fusion features are derived as follows: 


(12) \begin{align*} Local{\_}Fea&=MK{\_}Fea \otimes Local_{weight}\end{align*}

(iv) Combining global and local features

By concatenating $Global{\_ }Fea$ and $Local{\_ }Fea$ and feeding into $Conv_{1}$, the GLDF features ($GLDF{\_ }Fea$) are generated by the following: 


(13) \begin{align*} GLDF{\_}Fea&=Conv_{1}(cat(Global{\_}Fea, Local{\_}Fea))\end{align*}

Ultimately, $O_{embed}$ is transferred to $GLDF{\_ }Fea$ through a residual connection operation [38] to obtain the final multi-kernel dynamic fusion features $O_{MKFF}$, which transfers more information and reduces distortion. 


(14) \begin{align*} O_{MKFF} = O_{embed} \oplus GLDF{\_}Fea\end{align*}

where $\oplus $ is the broadcasting addition.

#### Downsampling residual feature embedding module

In Fig. 1D, to tackle the issues of high-dimensional features and low computational efficiency resulting from ineffective padding, we introduce a DRFE module, combining layer-by-layer downsampling, equal-length convolution, and residual connections. This module aims to efficiently compress the features, reducing dimensionality while retaining critical information.

First, we extract initial features on $O_{MKFF}$ through two convolutions. This operation captures the fundamental patterns and features present in $O_{MKFF}$, thereby facilitating the generation of more expressive feature representations. As follows: 


(15) \begin{align*} stem = Conv_{5}(Pad(Conv_{5}(Pad(O_{MKFF}))))\end{align*}

where a padding layer ($Pad$) ensures equal-length convolution that expands the receptive field, effectively captures long-range dependencies, and reduces information loss without increasing the parameters and computational complexity [39]. It also maintains the dimensions of the feature map, aiding subsequent layer-by-layer downsampling.

Then, we build the downsampling residual block (DR block). Details are as follows: 


(16) \begin{align*} X^{down}&=MaxPool_{3}(Pad(stem))\qquad\qquad\qquad\qquad \end{align*}

(17) \begin{align*} X^{DRFE}&=Conv_{5}(Pad(Conv_{5}(Pad(X^{down})))) \oplus X^{down}\end{align*}

By alternating between maximum pooling ($MaxPool_{3}$) and equal-length convolution, the spatial size of the feature map gradually reduces. Additionally, residual connections enable the network to retain important high-frequency information while reducing feature map size and maintaining the spatial consistency of feature maps [38]. Accordingly, this module employs residual connections to ensure the preservation of the original information integrity, thereby preventing the loss of significant features during the downsampling process. Three DR blocks are used in the DRFE module to obtain the required embedded features.

Furthermore, to learn a feature space where similar samples are closer and dissimilar samples are further apart, we introduce a contrastive learning loss function [40] ($\mathcal{L}_{cl}$) for representation optimization about DRFE features during training. For feature representations of two sequence $X^{DRFE}_{i}$ and $X^{DRFE}_{j}$: 


(18) \begin{align*} \mathcal{L}_{cl}=&\frac{1}{2}((1-K)D(X^{DRFE}_{i}, X^{DRFE}_{j})^{2}+ \nonumber \\ &K\{max(0, M-D(X^{DRFE}_{i}, X^{DRFE}_{j}))^{2}\})\end{align*}

where $K=0$ if two sequences belong to the same class, otherwise $K=1$. $D$ represents the Euclidean distance. $M$ is the margin. If $K=1$ and $D(X^{DRFE}_{i}, X^{DRFE}_{j})<M$, we optimize by moving them away from each other. To facilitate understanding, we draw a schematic diagram of the contrastive learning process, see Supplementary Figure S1.

#### Output module

Finally, the probability of a sample containing a m$^{6}$A site ($\hat{y}$) is calculated as follows (Fig. 1E): 


(19) \begin{align*} \hat{y}=FC_{Sigmoid}(Flatten(X^{DRFE}))\end{align*}

where $FC_{Sigmoid}$ denotes a fully connected layer with a $Sigmoid$ activation function. If $\hat{y}$ is less than 0.5, the sequence is classified as a negative sample (not containing m$^{6}$A site). Otherwise, it is classified as a positive sample (contains a m$^{6}$A site).

Here, we utilize the binary cross-entropy loss function as the objective function to minimize: 


(20) \begin{align*} \mathcal{L}_{bce}=-\frac{1}{N}\sum_{i=1}^{N}(y_{i}log(\hat{y}_{i})+(1-y_{i})log(1-\hat{y}_{i}))\end{align*}

where $N$ is the batch size, $y_{i}$ is the true label, and $\hat{y}_{i}$ is the predicted probability. Hence, the total loss function of the MFDm$^{6}$ARice is: 


(21) \begin{align*} \mathcal{L}_{total}=\mathcal{L}_{cl}+\mathcal{L}_{bce}\end{align*}

### Evaluation metrics

In this study, 5-fold cross-validation (5-CV) is adopted to evaluate the performance of MFDm$^{6}$ARice and state-of-the-art methods. For the benchmark dataset, we randomly select 80% as the training set and use the remaining 20% as the validation set to optimize model parameters. The final 5-CV result is the average of the results of five validation sets. Traditional evaluation metrics [41, 42], including accuracy (ACC), Matthew’s correlation coefficient (MCC), the area under the receiver-operating characteristic curve (AUC), and the area under the precision-recall curve (AUPR) are used to assess the performance of our proposed method and other methods.

## Results and discussion

This section introduces the experimental results, parameter analysis, and the effectiveness of each component of the MFDm$^{6}$ARice, accompanied by relevant visualizations and research.

### Comparison with state-of-the-art methods

To evaluate the performance of MFDm$^{6}$ARice, we compare it with SMEP and PEA-m6A, the leading methods for predicting m$^{6}$A sites in rice, using two types of datasets mentioned previously. Simultaneously, we choose DeepM6ASeq, a classic and well-established method for predicting m$^{6}$A in other species, as a comparison.

#### Cross-validation performance on benchmark dataset

The predictive performance of MFDm$^{6}$ARice is assessed on the benchmark dataset via 5-CV. As demonstrated in Table 2, MFDm$^{6}$ARice exhibits superior overall performance compared to the other methods. In particular, the model achieves an ACC of 0.8321, an MCC of 0.6225, an AUC of 0.9038, and an AUPR of 0.8201, exhibiting an 11.81% improvement in overall performance compared to the second-best method, DeepM6ASeq.

**Table 2:** A comparison of 5-CV performance for the various methods

Methods	ACC	MCC	AUC	AUPR
SMEP	0.7956 $\pm $ 0.0039	0.5370 $\pm $ 0.0163	0.8683 $\pm $ 0.0045	0.7601 $\pm $ 0.0100
PEA-m6A	0.7563 $\pm $ 0.0040	0.5028 $\pm $ 0.0038	0.8419 $\pm $ 0.0033	0.7095 $\pm $ 0.0082
DeepM6ASeq	0.8148 $\pm $ 0.0066	0.5747 $\pm $ 0.0168	0.8845 $\pm $ 0.0055	0.7864 $\pm $ 0.0113
**MFDm$^{6}$ARice**	**0.8321 $\pm $ 0.0036**	**0.6225 $\pm $ 0.0103**	**0.9038 $\pm $ 0.0028**	**0.8201 $\pm $ 0.0069**

Note: The best value of each index is bolded. The underlined means the second-best value.

Although the standard deviation (std) of MCC is not as optimal as PEA-m6A, the std of other metrics is superior to that of the comparative methods. These findings indicate that MFDm$^{6}$ARice demonstrates both notable performance and robustness.

#### Performance on independent test sets

To further illustrate the superiority and generalizability of MFDm$^{6}$ARice, we compare it with existing prediction methods on independent test sets, as presented in Table 3.

**Table 3:** The prediction performance of the various methods on two independent test sets

Datasets	Methods	ACC	MCC	AUC	AUPR
Same-species independent test set	SMEP	0.7940	0.5160	0.8687	0.7602
	PEA-m6A	0.7556	0.5000	0.8400	0.7074
	DeepM6ASeq	0.8031	0.5442	0.8755	0.7700
	**MFDm$^{6}$ARice**	**0.8243**	**0.6001**	**0.8941**	**0.8068**
Cross-species independent test set	SMEP	0.5056	0.0136	**0.5343**	0.5122
	PEA-m6A	0.5074	0.0150	0.5260	0.5069
	DeepM6ASeq	0.5126	**0.0337**	0.5261	**0.5218**
	**MFDm$^{6}$ARice**	**0.5147**	0.0318	0.5336	0.5200

Note: The optimal value is bolded. The second-best value is underlined.

In Table 3, our proposed method exhibits greater effectiveness than the comparative method concerning the same-species independent test set. Specifically, MFDm$^{6}$ARice improves ACC, MCC, AUC, and AUPR by 2.12, 5.59, 1.86, and 3.68%, respectively, exhibiting a 13.25% improvement in overall performance over the suboptimal method. Meanwhile, our proposed method is comparable to the cross-species independent test set. MFDm$^{6}$ARice has an overall performance slightly higher than the second-best method.

Notably, the results from the same-species independent test set in rice are comparable to those of the 5-CV results, indicating that our method exhibits robust and transferable performance on the same species. However, as expected, the performance of all methods diminishes markedly on the cross-species independent test set due to species and data type discrepancies. These findings highlight the challenges of developing multi-species m$^{6}$A prediction methods and identifying unseen data. Despite these difficulties, MFDm$^{6}$ARice outperforms the comparison methods in overall performance, suggesting its resilience to challenges and potential.

#### Extended application of MFDm$^{6}$ARice framework on maize

In addition, to evaluate the model’s utility and scalability on other species from the *Poaceae* family, we collect maize m$^{6}$A dataset from SMEP [29]. This dataset contains 11 150 positive samples and 22 300 negative samples. Like rice, the data in this dataset also have variable-length peak sequences. Then, we use the MFDm$^{6}$ARice framework tuned on the rice dataset to extend to this dataset. The processing and training of the MFDm$^{6}$ARice framework on this dataset are consistent with those on the rice dataset.

Supplementary Table S3 shows all results from the compared methods. As anticipated, the performance metrics of the models trained by directly applying the frameworks to the maize m$^{6}$A dataset are lower than those trained on the rice dataset. However, our method remains competitive. These findings indicate that MFDm$^{6}$ARice exhibits utility and potential for extension to other *Poaceae* plants.

### Parameter analysis

**Figure 2:**
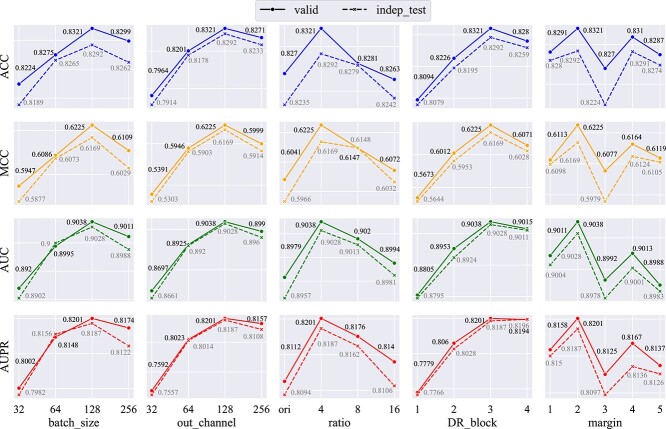
Performance of MFDm$^{6}$ARice on the validation set from benchmark dataset and the same-species independent test set with different values of batch size, out channel, ratio (reduction ratio), DR_block (downsampling residual block), and margin based on 5-CV. The solid lines and black values are the validation set results. The dashed lines and gray values are the independent test set results.

To evaluate the influence of main hyperparameter settings on model performance, we conduct a hyperparameter sensitivity analysis [43, 44] encompassing batch size, output channels, reduction ratio (in GLDF’s Local dynamic fusion), DR block (in DRFE), and margin through 5-CV. The hyperparameters ultimately used in this study are summarized in Table 4. At the same time, we further explore the impact of these hyperparameter changes on the validation set and the same-species independent test set. The results are shown in Fig. 2. Supplementary Section S3, Supplementary Tables S4 and S5 provide results and analyses of additional hyperparameters, such as the learning rate and the number of layers in the convolutional blocks. We also offer a hyperparameter selection process in Supplementary Section S4.

**Table 4:** The optimal hyperparameter settings of MFDm$^{6}$ARice

Hyperparameter	Setting
Batch size	128
Out channel	128
Ratio	4
DR block	3
Margin	2

Note: The ratio is the abbreviation for reduction ratio. DR block is the downsampling residual block.

#### The impact of various hyperparameter settings on validation set

Examining the validation set results (solid line) in Fig. 2, we observe the following:

(i) Output channels: The number of output channels strongly affects model performance. As seen in the second column of Fig. 2, changing the out_channel from 32 to 128 yields differences in ACC, MCC, AUC, and AUPR by 3.57, 8.34, 3.41, and 6.09%, respectively. Increasing output channels allows the network to capture more complex features, enhancing its capacity to learn intricate patterns in the data. However, further increasing output channels (e.g. out_channel = 256) can lead to overfitting, reducing performance.
(ii) Number of DR Blocks: The number of DR blocks also significantly impacts performance. In the fourth column of Fig. 2, the performance metrics (ACC, MCC, AUC, and AUPR) vary by 2.27, 5.52, 2.33, and 4.22%, respectively, between DR_block=1 and DR_block=3. DR blocks modify the network structure by controlling feature map resolution, computational complexity, and information flow through downsampling and residual connections. Moderate information compression enhances performance, but excessive compression (e.g. DR_block=4) limits feature information, ultimately reducing performance.
(iii) Batch size: Batch size has a noticeable, though smaller, effect. As shown in the first column of Fig. 2, changing the batch size from 32 to 128 results in performance differences of 0.97, 2.78, 1.18, and 1.99% in ACC, MCC, AUC, and AUPR, respectively. Larger batch sizes offer more accurate gradient estimates, stabilizing convergence. However, very large batch sizes (e.g. batch size = 256) may cause the model to converge to suboptimal solutions, leading to a slight performance decrease.
(iv) Reduction ratio and margin: These two hyperparameters have minimal impact on performance, suggesting that the model is relatively insensitive to them compared to the more influential settings above.

#### Comparing results for different hyperparameter settings on validation set and independent test set

By comparing the results of the validation set and independent test set (dashed line) based on 5-CV, we observe that the general trends and impacts of different hyperparameter settings are closely consistent between the validation set and the independent test set. It shows that the hyperparameters selected by cross-validation in this work are effective. What’s more, there is little difference between the validation and independent test set results. This highlights the model’s strong generalization ability. These consistencies show that our model learns to generalize effectively and to remember the training data, ensuring its practical application in real-world scenarios.

### Effectiveness of the MKFF module’s functions

MFDm$^{6}$ARice, proposed in this work, designs a multi-kernel feature extraction with a GLDF module, MKFF. The module begins with extracting multi-kernel features and then fuses them through a global–local dynamic attention mechanism. To assess the efficacy of the MKFF module, we conduct an ablation study and effectiveness evaluation on two key functions: the multi-kernel feature extraction and the GLDF. Details are as follows.

#### Validity of multi-kernel feature extraction function

**Figure 3:**
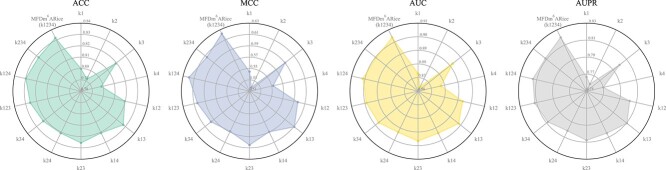
Performance of MFDm$^{6}$ARice on the benchmark dataset with different kernel feature combinations. k1 means only using kernel1 features. k12 represents the features combination of kernel1 and kernel2, k123 denotes the features combination of kernel1, kernel2, and kernel3, and so forth. See (2), (3), (4), and (5) for details.

In the multi-kernel feature extraction function, we use (2), (3), (4), and (5) to obtain the multi-kernel features (k1234) of kernel1 ($k1$), kernel2 ($k2$), kernel3 ($k3$), and kernel4 ($k4$). To verify the effectiveness of k1234, we set up a comparative experiment with different kernel feature combinations, as shown in Fig. 3. In this experiment, k1 means only using kernel1 features. k12 represents the features combination of kernel1 and kernel2, k123 denotes the features combination of kernel1, kernel2, and kernel3, and so forth. Note that, except for the different feature combinations, the remainder of the model framework remains unaltered in this comparative experiment.

As evidenced in Fig. 3, the overall performance of the combined features exceeds that of a single kernel feature. The k1234 used in this work is optimal, i.e. four-kernel features combination. The performance of the three-kernel feature combination is superior to that of the two-kernel feature combination. It shows that, with the addition of different kernel features, the model can capture feature information of varying receptive fields, obtain more diverse feature maps, and provide more comprehensive information input for subsequent layers. It enhances model performance and demonstrates the efficacy of multi-kernel features.

#### Validity of global–local dynamic fusion function

To assess the effectiveness of the GLDF function, we design two types of comparative experiments: internal and external comparisons.

For the internal comparisons, we construct four GLDF variants:

- Replacing the GLDF module with concatenation (cat).
- Replacing the GLDF module with addition (add).
- Removing local dynamic fusion (global).
- Removing global dynamic fusion (local).

For external comparisons, we use other widely adopted feature fusion techniques: SE [37], ECA [36], and CBAM [45]. These methods are often employed to strengthen feature representation by focusing on specific feature channels or spatial locations. By replacing GLDF with these techniques, we can evaluate GLDF’s relative effectiveness.

**Figure 4:**
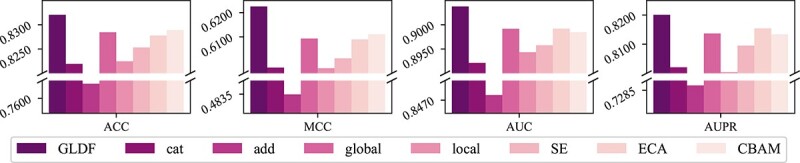
Comparative ablation study of the GLDF function. GLDF represents global–local dynamic fusion; cat is concatenation; add is addition; global means only global dynamic fusion; local means only local dynamic fusion. SE, ECA, and CBAM are popular modules used for feature fusion.

The results in Fig. 4 demonstrate that the GLDF module generally outperforms both internal and external comparison models, underscoring its effectiveness in dynamically fusing multi-kernel features. Concurrently, we observe the following:

(i) Superiority over concatenation and addition: The GLDF, SE, ECA, and CBAM modules perform better than simple concatenation or addition. Even the isolated components of GLDF (global and local dynamic fusion) outperform concatenation and addition. It suggests that simple fusion techniques like concatenation or addition fail to account for the distinct importance of each kernel feature map, which can impair model performance.
(ii) Addition versus concatenation: Adding feature maps yields worse results than concatenation. Element-wise addition of feature maps from different kernels can lead to information loss and reduce the complementarity between feature maps, resulting in less effective feature interaction and weaker feature representation.
(iii) Importance of Global Features: Global fusion contributes more to performance than local fusion. In classification tasks, global features often carry more importance than local details. Global channel dynamic fusion captures overall statistical information through global average pooling, enabling a more adaptive and comprehensive adjustment of each channel’s importance. In contrast, local spatial dynamic fusion focuses on adjusting weights within smaller areas, constrained by the receptive field, limiting its ability to capture global context.
(iv) Effective of Combined Global and Local Fusion: Despite the global fusion being more effective than the local fusion on its own, combining both global and local dynamic fusion (GLDF) still outperforms the external comparison models. This result suggests that blending global and local information enhances the model’s ability to capture and represent features. This observation is further supported by the results of ECA and CBAM in Fig. 4, which also performs better than SE, a model that only uses global information.
(v) Comparing Global Fusion and GLDF: While the global-only variant and the GLDF module yield similar overall performance, incorporating local dynamic fusion provides added benefits, especially for sequences of varying lengths. By analyzing the impact of local fusion on different sequence lengths, we find that integrating local information corrects more false negatives and improves the model’s ability to identify true positives. This effect is particularly pronounced in shorter sequences (200–400 length range), where the model shows fewer false positives and false negatives.

These findings underscore the value of combining global and local features for a more robust feature representation, enhancing the model’s capability to capture nuanced details across sequences. Further details and supporting figures for this analysis are available in Supplementary Section S5, Figures S2 and S3.

### Ablation study of MFDm$^{6}$ARice

To ascertain the individual role of each component in the proposed MFDm$^{6}$ARice, we conduct a series of ablation experiments by building several model variants, as shown below:

- **MFDm$^{6}$ARice without contrastive learning (MFDm$^{6}$ARice w/o CL)**: This variant removes the feature representation optimization based on contrastive learning in the DRFE module.
- **MFDm$^{6}$ARice without MKFF (MFDm$^{6}$ARice w/o MKFF)**: This variant removes the MKFF module, which is used to extract and globally-locally dynamically fuse multi-kernel features.
- **MFDm$^{6}$ARice without DRFE (MFDm$^{6}$ARice w/o DRFE)**: This variant removes the DRFE module, which balances computational efficiency and feature expressiveness.

Table 5 shows that MFDm$^{6}$ARice performs better or similarly across all variants. Removing any component impacts performance, demonstrating that the components of the MFDm$^{6}$ARice framework work together to enhance performance. Key observations include:

(1) The performance of MFDm$^{6}$ARice without MKFF is lower than that of MFDm$^{6}$ARice without DRFE, indicating that MKFF has a greater contribution than DRFE. It suggests that the feature enhancement method, which extracts and dynamically fuses multi-kernel features with global–local attention, is effective in improving model performance for rice m6A site prediction.
(2) While the DRFE module contributes less than MKFF, it still impacts the overall performance of MFDm$^{6}$ARice. Combined with the results in the penultimate column of Fig. 2, it is clear that appropriate downsampling reduces memory overhead while effectively improving model performance.
(3) The results for MFDm$^{6}$ARice without CL show that contrastive learning optimization of feature representation contributes the least but still offers slight improvement. It may be because contrastive learning is applied to the same feature source (a single modality), which limits the gain, as verified by experiments in CGIP [46].

**Table 5:** The performance of MFDm$^{6}$ARice and its variants using 5-CV

Variants	ACC	MCC	AUC	AUPR
**MFDm$^{6}$ARice**	**0.8321**	**0.6225**	**0.9038**	**0.8201**
MFDm$^{6}$ARice w/o CL	0.8317	0.6186	0.9017	0.8169
MFDm$^{6}$ARice w/o MKFF	0.7305	0.4358	0.8079	0.6496
MFDm$^{6}$ARice w/o DRFE	0.7873	0.5174	0.8535	0.7339

Note: the optimal value of each metric is bolded.

**Figure 5:**
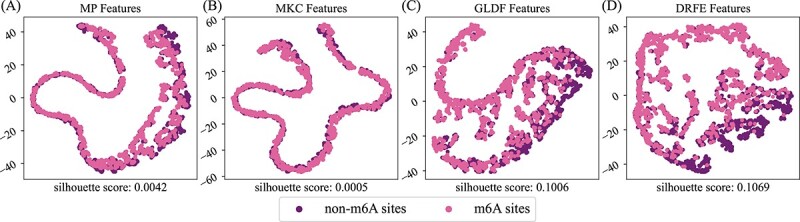
t-SNE visualization of key component features of MFDm$^{6}$ARice. (A) MP Features mean max-length padded sequence features. (B) MKC Features mean multi-kernel concatenated features that are input to the GLDF function. (C) GLDF Features are global–local dynamic fusion features. (D) DRFE Features are downsampling residual feature embedding features. The silhouette score (the bigger, the better) is a quantitative assessment.

To more intuitively observe the effectiveness of each component of MFDm$^{6}$ARice, t-SNE (t-distributed stochastic neighbor embedding) [47] is used to visualize the distribution of max-length padded sequence features (MP Features), multi-kernel concatenation features (MKC Features, input of GLDF function), global–local dynamic fusion features (GLDF Features), and downsampling residual feature embedding features (DRFE Features) by reducing their features to 2D, respectively (presented in Fig. 5)). Simultaneously, we compute the silhouette score [48] (the higher, the better) as a quantitative assessment of the clustering effect. Figure 5A shows that the max-length padded features obtained by the initial label encoding and padding have almost no classifying ability. Compared with the silhouette score in Fig. 5B, the features obtained by the GLDF function (Fig. 5C) have greatly improved the classification performance, consistent with the results in Fig. 4. However, direct concatenation of the extracted multi-kernel features has a lower silhouette score than max-length padded sequence features. The possible reason could be that direct concatenation leads to feature redundancy and inconsistent scaling of features in the same channel, which may reduce the model’s effectiveness. To sum up, the utility and necessity of the GLDF function have been re-affirmed. Based on the GLDF features, we use the DRFE module to obtain the embedded downsampled residual features. Its performance has been improved to some extent, revealing again the DRFE module’s effectiveness (Fig. 5D).

### Case study

To further explore the practical value of the model, we conduct a case study on the motifs learned by MFDm$^{6}$ARice. The gradient reflects the model’s sensitivity to each position of the input data, indicating which positions contribute most to the prediction results. To identify motifs using MFDm$^{6}$ARice, we first calculate the gradient of the sequence. Then, we apply a fixed sliding window to capture high-contribution regions. These high-contribution regions are mapped to the sequence to obtain the corresponding local sub-sequences. Finally, MEME [49] performs multiple sequence alignment on these local sub-sequences, obtaining the motifs learned by MFDm$^{6}$ARice. Using TOMTOM [49], these learned motifs are matched with known motifs (with plant- or rice-related databases), yielding 40 unique highly significant motifs (*P*-value < 0.001) (see Supplementary Table S6). Supplementary Figure S4 shows the sequence logo diagrams of some significantly learned motifs. It demonstrates that MFDm$^{6}$ARice can capture the true characteristics of m$^{6}$A modification.

Interestingly, these highly matched known motifs are involved in rice biotic and abiotic stresses, growth, and development, similar to rice m$^{6}$A modification. For example, the NAC-GCM regulates rice response to drought and nutrient stress by controlling genes involved in cell wall remodeling and stress signaling [50]. The EIL negatively modulates salt stress tolerance through the ethylene signaling pathway, affecting root and coleoptile development [51]. The TCP integrates endogenous signals and environmental cues to adjust rice growth responses under stress conditions, enhancing survival in adverse environments [52]. It suggests that m$^{6}$A modification may be related to regulating gene expression associated with these motifs.

In addition, we have identified several novel motifs that did not meet the significance threshold for matching in known databases. See Supplementary Table S7. Notably, some novel motifs show sequence similarity to highly significant motifs, such as MEME-20, MEME-53, and MEME-71, suggesting they may be potential m$^{6}$A-related motifs for rice. These potential novel motifs could serve as candidates for further experimental validation to investigate their roles in rice regulatory networks. By combining these known and potential novel motifs, we can further explore the regulatory role of m$^{6}$A modification in rice, ultimately contributing to precision breeding and improvement.

## Conclusion

m$^{6}$A is the most common post-transcriptional modification in eukaryotic RNA, regulating rice growth, development, and stress responses. Predicting m$^{6}$A sites in rice helps understand epigenetic regulation and supports precision breeding. This paper proposes MFDm$^{6}$ARice, a method for predicting rice m$^{6}$A sites using multi-kernel feature extraction, GLDF, and downsampling residual embedding, addressing issues of feature sparsity, high dimensionality, and low computational efficiency caused by max-length padding. After encoding and padding, MFDm$^{6}$ARice extracts and combines multi-kernel features to reduce sparsity and enhance data representation, while downsampling reduces feature map size and complexity. Results from the benchmark and independent datasets show that MFDm$^{6}$ARice outperforms existing methods in accuracy, robustness, and generalization. It is also scalable to maize. Ablation studies and t-SNE visualizations confirm the effectiveness of dynamic fusion and downsampling in improving feature representation and m$^{6}$A site prediction accuracy.

Despite its promising performance, MFDm$^{6}$ARice presents several limitations that warrant attention. First, considering the variable-length sequences, simplicity, and computational efficiency, we use label encoding with maximum length padding to process RNA sequences. However, we recognize that this encoding method may overlook the chemical properties of nucleotides and potential sequence dependencies within RNA sequences. Therefore, in future work, we aim to explore alternative encoding methods based on biochemical properties and embedding-based representations to enhance the feature representation of variable-length sequences. Additionally, we discuss the potential impacts of these alternative encoding methods on model interpretability and performance in Supplementary Section S6. These could provide a more comprehensive encoding method, thereby addressing current limitations.

While the DRFE module effectively optimizes feature space compression, reducing model parameter count and computational consumption, it still presents relatively high computational costs and model complexity compared to simpler architectures such as those without DRFE, MLP, or stacked convolutional layers. These differences in efficiency and complexity are detailed in Supplementary Section S7 and Supplementary Table S8. As a result, the high computational demands may limit the model deployment in resource-constrained environments, such as large-scale agricultural applications. Therefore, in future work, we aim to address these challenges, including reducing storage and memory consumption and exploring techniques such as model pruning, quantization, and lightweight deployment frameworks. Detailed discussions can be found in Supplementary Section S8.

The dataset used in this study is specific to rice, which may introduce biases and limit the model generalizability. Although MFDm$^{6}$ARice demonstrates scalability with maize, it is not designed for multi- or cross-crop applications, limiting its broader utility. In contrast, methods like those proposed by [31, 53, 54] aim to predict methylation sites across multiple species. Future research could focus on developing a broad-spectrum m$^{6}$A prediction model that accommodates various crops or species, enhancing its applicability. Additionally, inherent biases in the dataset, such as the under-representation of specific sequence types or environmental factors influencing m$^{6}$A modifications, should be addressed in future studies. Incorporating more diverse datasets or integrating omics data from different conditions could help provide a more comprehensive biological context and improve model performance. For a more concrete plan, see Supplementary Section S9.

In summary, future efforts will focus on expanding the model’s applicability to multi-crops prediction, integrating more detailed molecular features, and refining the rice m$^{6}$A dataset, which collectively will enhance both the accuracy and generalizability of m$^{6}$A site predictions.

**Key Points**

- We propose an end-to-end rice m$^{6}$A site prediction learning network, MFDm$^{6}$ARice, which can effectively learn the complete information of max-length padded sequences.
- To reduce feature sparsity caused by max-length padded sequences and effectively transfer the features extracted by multi-kernel, we design the MKFF module with a multi-kernel feature extraction and a GLDF mechanism that enriches and enhances feature representation and suppresses useless information.
- To solve the high-dimensional features and low computational efficiency caused by invalid padding, we introduce the DRFE module to efficiently compress features through layer-by-layer downsampling and residual connections, ensure valid information transfer, and improve computational efficiency.
- Extensive comparative, ablation experiments, and visualization studies demonstrate the superior performance of MFDm$^{6}$ARice and the rationality and effectiveness of the MKFF and DRFE modules.

## Supplementary Material

supplementary_materials_bbae647

supplementary_tables_bbae647

## Data Availability

The data set and source code can be downloaded from https://github.com/zhlSunLab/MFDm6ARice.
