# Supplementary material for: Multi-kernel feature extraction with dynamic fusion and downsampled residual feature embedding for predicting rice RNA N6-methyladenine sites
Source: Brief Bioinform. 2024 Dec 14;26(1):bbae647. doi: 10.1093/bib/bbae647 (PMC11646132; doi:10.1093/bib/bbae647)
Supplement: supplementary_materials_bbae647 [file supplementary_materials_bbae647.docx]

**SUPPLEMENTARY MATERIALS**

**Multi-kernel feature extraction with dynamic fusion and downsampled residual feature embedding for predicting rice RNA *N*^6^-methyladenine sites**

Mengya Liu^1^, Zhan-Li Sun^2, *^, Zhigang Zeng^3^, Kin-Man Lam^4^

1. School of Computer Science and Technology, Anhui University, Hefei, 230601, China
2. School of Electrical Engineering and Automation, Anhui University, Hefei, 230601, China
3. School of Artificial Intelligence and Automation, Huazhong University of Science and Technology, Wuhan, 430074, China
4. Department of Electronic and Information Engineering, The Hong Kong Polytechnic University, Hong Kong, China

**Corresponding author:**

Zhan-Li Sun, Email: zhlsun2006@126.com

**Contents**

**S1.** Various Padding Lengths Experiment Analysis

**S2.** Selection of Kernel Size and Experimental Analysis of Different Kernel Sizes

**S3.** Results and Analysis of Different Learning Rates and Number of Layers in Convolutional Block

**S4.** Hyperparameter Selection Process

**S5.** Comparing Global Fusion and GLDF

**S6.** Brief Discussion of Alternative Encoding Methods

**S7.** Computational Efficiency and Model Complexity of DRFE with Simple Architecture

**S8.** Discussion and Future Improvements of Model Deployment in Computationally Constrained Environments

**S9.** A More Concrete Plan for Addressing Limitations of The Dataset in Future

**Algorithm S1.** MFDm6ARice for rice m6A sites prediction.

**Figure S1.** Diagram of contrastive learning optimization process.

**Figure S2.** Sequence length distribution.

**Figure S3.** The difference between the confusion matrices of GLDF and global variants under different sequence length distributions.

**Figure S4.** Visualization of some significant motifs learned by MFDm6ARice.

**Reference**

**Supplementary Tables in the supplementary_tables.xlsx document:**

**Table S1.** Results of various padding lengths

**Table S2.** Results of different kernel sizes

**Table S3.** A comparison of 5-fold cross-validation performance for the various methods on maize

**Table S4.** Results of various learning rates

**Table S5.** Results of various number of layers in convolutional block

**Table S6.** Motifs learned by MFDm6ARice

**Table S7.** High-contribution novel motifs learned by MFDm6ARice

**Table S8.** Comparison of computational efficiency and model complexity between DRFE and simple architecture

1. **Various Padding Lengths Experiment Analysis**

To verify the model's performance under different sequence padding lengths, we conduct experiments on padding lengths of 400, 600, 800, 1000, and 1200, and **Supplementary Table S1** shows the results. The experimental results show that the overall performance of the padding length of 800 is the best, and all metrics reach the highest values. The specifics are as follows:

When the padding length is 400, the model performance is relatively low. It shows that the 400 padding length lacks to provide sufficient information, which may impede the model's ability. In addition, a shorter padding length may lead to inadequate data, thus affecting the classification and prediction performance of the model.

After increasing the padding length to 600, the model exhibits enhanced performance. The padding length of 600 shows that by increasing the padding length, the model can obtain more information and thus better understand the input features. However, compared with longer padding lengths, the model still does not achieve the best performance at 600, indicating that the amount of sequence information may still be insufficient.

With a padding length of 800, the model achieves the best performance. The reason for choosing a padding length of 800 is that the maximum sequence length of the dataset is 799, and 800 can fully cover the maximum sequence length without generating too much redundancy. Length 800 provides enough information while avoiding the noise caused by redundancy. Therefore, with a length of 800, the model can best capture the information in the sequence and achieve the best overall performance.

When increasing the padding length to 1000 and 1200, the model performance remains stable, although slightly lower than the performance of 800. The results show that, after increasing the padding length, the model can still effectively deal with the problem of feature sparsity. Although the input sequence may contain additional padding information, this does not significantly impact the model, indicating that the model is highly robust in dealing with redundant information.

In conclusion, the 800 padding length represents a reasonable and efficient choice, providing sufficient information while avoiding the potential issues associated with a shorter length. At the same time, it verifies the model's advantages in dealing with sparse features and redundant information, making it show good generalization ability when dealing with different input lengths.

1. **Selection of Kernel Size and Experimental Analysis of** **Different Kernel Sizes**

1, 3, and 5 are convolution kernel sizes widely used for feature extraction in deep learning. Especially in the Inception [1] structure, multiple convolution kernel sizes (e.g., 1, 3, and 5) are a common practice for parallel extract features of varying scales. This design can capture local details and a larger range of contextual information, thereby enhancing the diversity of feature expression and the robustness of the model.

To further verify the rationality of our choice, we conduct additional experiments evaluating kernel size combinations other than the original (1, 3, 5). Specifically, we experiment with larger kernel sizes (such as 7 and 9) to examine the model’s sensitivity to changes in receptive field sizes and to provide insights into the feature extraction process. **Supplementary Table S2** summarizes the results.

The experimental results show that alternative kernel size combinations (1, 3, 7 and 1, 3, 9) yield overall performance slightly lower than the original (1, 3, 5) combination. The performance difference, however, is minor (less than 1%), indicating that the model is relatively robust to changes in kernel size combinations.

From the results, we observe that:

**(a) Robustness to Receptive Field Changes**: The MFDm^6^ARice model demonstrates a certain degree of robustness to varying receptive field sizes, as performance remains consistent across different kernel size combinations.

**(b) Trade-off Between Receptive Field and Computational Efficiency**: While larger kernels (e.g., 7 or 9) provide a wider receptive field, the increased computational burden does not bring proportional improvements in performance. For instance, despite the augmented receptive field, the 1, 3, and 9 combination exhibits slightly diminished performance relative to the original 1, 3, and 5.

**(c) Effectiveness of the Original Combination (1, 3, 5)**: The results confirm that the 1, 3, 5 combination achieves an optimal balance between model performance and computational efficiency. This combination provides sufficient receptive field coverage for effective feature extraction without incurring unnecessary computational costs.

The results from these additional experiments validate our choice of the (1, 3, 5) kernel size combination. This configuration allows the MFDm^6^ARice model to achieve superior performance while maintaining efficiency, highlighting its adaptability and effectiveness in feature extraction across varying receptive field sizes. This robustness further underscores the model’s practical utility and potential application across different settings without the need for extensive tuning of kernel sizes.

1. **Results and Analysis of Different Learning Rates and Number of Layers in Convolutional Block**

**(i) Learning rate**

We select three commonly used initial learning rates of 0.1, 0.01, and 0.001 to conduct experiments to observe the model's performance under different update steps. These three learning rates represent large, medium, and small learning rate settings, respectively.

From the **Supplementary Table S4**, the learning rate of 0.001 achieves the best performance, with an ACC of 0.8321, an AUC of 0.9038, and an AUPR of 0.8201, which shows that the model has strong robustness and stable convergence characteristics under this learning rate. In contrast, although the learning rate of 0.01 also performs stably (ACC of 0.7769 and AUC of 0.8479), it is slightly inferior to 0.001 in various metrics, indicating that the learning rate converges slowly and may require longer training time.

The learning rate of 0.1 performs poorly in all metrics (ACC of 0.6667 and AUC of 0.5) and is even much lower than other configurations. It may be because the excessively high learning rate caused the model parameters to update too much, which in turn caused unstable convergence or trapped in local minima.

Finally, we choose 0.001 as the initial learning rate, commonly employed by the Adam optimizer, which exhibits the optimal performance in this experiment. This configuration enables the model to attain a balance between high performance and stability.

**(ii) Number of layers in convolutional blocks**

We test the effects of different numbers of layers, i.e., 1, 2, and 3, in the convolutional block.

As illustrated in **Supplementary Table S5**, the 1-layer convolutional block structure exhibits the best performance. Specifically, the ACC is 0.8321, the AUC is 0.9038, and the AUPR is 0.8201, showing that the model can effectively learn and express input features.

The 2-layer convolutional block structure performs second best (ACC is 0.8215, AUC is 0.8890), slightly lower than the 1-layer convolutional block configuration, but performs well in performance and stability. This result shows that increasing the number of layers in convolutional blocks can improve the model's feature extraction ability to a certain extent. However, the effect is constrained beyond a certain number of layers.

The 3-layer convolutional block structure shows relatively low accuracy and AUC (ACC is 0.7975, AUC is 0.8694) because the more complex model structure increases the risk of overfitting and the computational overhead. Therefore, too many convolutional block layers may lead to performance degradation in this task.

Overall, the 1-layer convolutional block structure reduces the demand for computing resources while ensuring high performance, providing higher efficiency for practical applications.

Based on the above experimental results, we finally choose the configuration of a learning rate of 0.001 and 1 convolutional block. This combination achieves the best results in terms of accuracy, AUC, and AUPR, taking into account performance, stability, and computational efficiency.

1. **Hyperparameter Selection Process**

To ensure the reliability of the results and the best performance of the model, the selection of hyperparameters follows the following process:

**Step 1. Determine the hyperparameters to be adjusted**

According to the characteristics of the model and the task requirements, select a set of hyperparameters to be adjusted, such as learning rate, batch size, and number of output channels.

**Step 2. Set the initial value and search range**

The initial value and search range of each hyperparameter are set based on literature and experience, such as:

Learning rate: 0.1, 0.01, and 0.001 are selected as candidate values.

Batch size: 32, 64, 128, and 256 are selected as candidate values.

Number of output channels: 32, 64, 128, and 256 are selected as candidate values.

**Step 3. Manually adjust hyperparameters**

During the parameter adjustment process, a step-by-step adjustment method is adopted, fixing other hyperparameters each time and focusing on adjusting one hyperparameter, such as:

Step 3.1: Adjust the learning rate, fix the batch size and the number of output channels, and select the optimal learning rate.

Step 3.2: After determining the optimal learning rate, adjust the batch size and select the optimal value.

Step 3.3: Finally, adjust the number of output channels and select the best configuration.

**Step 4. Result analysis and summary**

For each candidate value of the hyperparameter, multiple rounds of experiments are conducted, and the impact of each setting on the model performance is evaluated through cross-validation. Finally, the best combination is selected.

In addition, when the number of adjusted hyperparameters is large or the training time is long, manual parameter adjustment may lead to excessively high computational costs. Automatic parameter adjustment methods can efficiently find the optimal configuration in a short time. For example, bayesian optimization in the hyperopt package [2] can be an effective choice. Although automatic parameter adjustment methods may be more efficient in some cases, in this study, based on the controllability and simplicity of the experiment, we choose manual parameter adjustment as the main strategy.

1. **Comparing Global Fusion and GLDF**

To more comprehensively analyze the difference between global and GLDF, we conduct the following experiments:

(i) Considering the variable length of rice m^6^A sequences, we first analyze the sequence length distribution, as shown in **Supplementary Figure S2**. The sequence length distribution is 200-800, mainly concentrated in 200-400.

(ii) We then categorize the sequences into different length groups: all sequences, sequences of length 200-400, 400-600, and 600-800. For each distribution of sequence lengths, we calculate the differences between the confusion matrices of the GLDF and global variants to observe their performance differences.

As shown in **Supplementary Figure S3**, after incorporating local information from local dynamic fusion, although introducing some noises, resulting in reduced model attention to negative samples, more false negative samples are corrected overall, enhancing the model's ability to identify true positive samples. It is particularly evident in the 200-400 sequence length class, with improvements in false negatives and true positives and a reduction in false positives. It indicates the usefulness and necessity of incorporating local information for variable-length sequences, especially for shorter sequences.

Therefore, while the performance of global variant and GLDF may not differ significantly, effectively extracting and utilizing local information is essential to improving the model's understanding of sequence details and further enhancing its performance.

1. **Brief Discussion of Alternative Encoding Methods**

**Encoding based on biochemical properties:** Encoding methods based on biochemical properties can take into account the physicochemical properties of each nucleotide (such as the number of hydrogen bonds, hydrophilicity, hydrophobicity, etc.), provide more comprehensive and detailed molecular-level information, and thus help the model better understand the interactions and structural properties between nucleotides in a biological sense. Since m^6^A involves methyl groups at the RNA molecular level, this encoding method may improve the model's understanding of this biological behavior, thereby improving the accuracy of the m^6^A site prediction task.

**Encoding based on embedding representations**: Using a pre-trained RNA embedding model, the complex structure and functional information of the RNA sequence can be better captured by learning the contextual relationship of each nucleotide in the sequence. For example, based on methods such as BERT [3], DNABERT [4], and Word2Vec [5] in natural language processing, by fine-tuning the sequence of rice m^6^A-specific tasks, a higher-level representation is generated for each nucleotide in RNA, revealing the complex relationship between nucleotides and improving the understanding of complex biological features. This method of understanding the interactions between nucleotides through context helps to improve the interpretability of the model. Furthermore, the embedded representation has the potential to enhance performance by enabling the model to capture more intricate sequence patterns in RNA, which may prove challenging to achieve through traditional label encoding.

1. **Computational Efficiency and Model Complexity of DRFE with Simple Architecture**

To observe the computational efficiency and model complexity of the DRFE module compared with other simple architectures, we supplement multiple sets of experiments to compare the performance of the model containing the DRFE module with three other simplified architectures (i.e., no DRFE module, DRFE replaced by MLP, and DRFE replaced by CNN). We record the training time to obtain the final model on the benchmark dataset (59,536 samples), the prediction time on the independent test set of the same species (14,886 samples), the model complexity (FLOPs, floating point operations), the number of parameters, and the GPU memory usage to quantify the computational consumption and complexity of each model.

From **Supplementary Table S8**, we can find that:

(a) Computational cost and model complexity: The model with the DRFE module shows the high training time, prediction time, FLOPs, and GPU memory usage. This is mainly attributed to the fact that the DRFE module increases the computational requirements of the model while compressing the feature space by introducing additional convolutions, pooling, padding, and residual connections. DRFE aims to enhance the expressiveness of the model by extracting and processing features more efficiently, but this enhanced performance also brings higher computational overhead.

(b) Parameter quantity: Although the DRFE module increases computational cost, it does not significantly increase the number of parameters by effectively reducing the dimension and embedding the features. In fact, the number of parameters of the model with DRFE is second only to the model where DRFE is replaced by MLP architecture, indicating that DRFE can improve the model’s performance while maintaining fewer parameters. It is because the DRFE module compresses the input feature space through residual connection and feature embedding technology, reducing the redundant information that needs to be processed.

(c) Performance improvement: The model with the DRFE module performs best, with each improved by 5-10% compared with the other three architectures. This result can be attributed to the DRFE module’s feature compression and information flow, which enables the model to learn more abstract and helpful features more effectively. Therefore, DRFE significantly improves the model's performance despite the high computational cost.

1. **Discussion and Future Improvements of Model Deployment in Computationally Constrained Environments**

Our final prediction model, MFDm^6^ARice, is very compact, with a size of only 2.91MB, which is very friendly to resource-constrained environments. It means that on most hardware devices, the model can be quickly loaded and inferred without occupying a lot of memory. However, in actual deployment, the packaged runtime environment (for example, packaged using Conda or Docker) may have higher resource requirements. Specifically, the packaged Conda environment in this work is about 2GB, while the Docker image is 10GB because it contains a complete Ubuntu system. Although most of the space in these environments is not occupied by itself, they still affect the performance and storage requirements when deployed to devices with limited computing resources.

Therefore, although the model itself is small, the packaged runtime environment may bring certain storage pressure. At the same time, **Supplementary Section S7** also shows that the performance improvement brought by DRFE is accompanied by higher computational overhead, which makes the model face computational resource challenges in large-scale agricultural applications. Therefore, in future work, if we need to deploy the model to an environment with limited computing resources, such as low-power embedded devices or edge computing nodes, we can consider (a) reducing storage and memory consumption by removing unnecessary dependencies, compressing the Conda environment, or optimizing the Docker image. (b) Combining model optimization techniques such as model pruning, quantization, and using lighter frameworks (such as TorchScript [6] or ONNX Runtime) to reduce the size of the deployment package, further reduce memory usage and computing burden, and make the model more suitable for deployment on low-resource devices.

1. **A More Concrete Plan for Addressing Limitations of The Dataset in Future**

In this work, the specificity of predicting rice m^6^A sites is a limitation. We plan to take a series of specific measures in future studies to expand and enhance the dataset, thereby improving the model's generalization ability and reducing potential bias. Details are as follows:

(1) Expand the dataset:

Currently, the plants in the dataset are mainly concentrated in the specific rice species. To improve the generalization ability of the model, we plan to obtain more extensive m^6^A data from the following databases:

(a) m^6^A-Atlas [7]: This is a broad m^6^A database that covers m^6^A data of multiple plant species. By integrating the m^6^A modification data of multiple plant species, the model can learn the common features and differences between different plant species, thereby improving its cross-species prediction ability.

(b) m^6^A-Seq dataset: We will build a diverse training set through public RNA-seq data (such as the plant m^6^A dataset in the GEO database [8] ) combined with m^6^A sequencing data of multiple plants under different stress conditions.

(2) Alleviate data bias:

In the existing dataset, we have noticed that there may be some bias problems in the current dataset. For example, some sequence lengths account for too low a proportion in the dataset (as shown in the attached figure), and the stress type samples are single (heat shock), which may cause the model's prediction effect in these cases not to be as good as in other types of samples. Therefore, in future work:

(a) Unbalanced sequence length:

(i) Balanced sampling: Balanced sampling of sequences of different lengths that the proportion of long and short sequences in the training data is reasonable.

(ii) Data enhancement: Use data enhancement methods (such as random cropping, sequence expansion, etc.) to deal with the deviation of sequence length.

(b) Sample imbalance of stress types:

(i) Collect data under multiple stress conditions: Considering the differences in the performance of plants under different environmental stress conditions, we will use databases such as m^6^A-Atlas to obtain m^6^A data of plants under various stresses.

(ii) Synthetic data: Balance the dataset by generating synthetic data of minority stress types. For example, SMOTE [9] or generative adversarial networks (GANs) [10] generate m^6^A data under different stress types to expand the training set.

(iii) Weighted loss function: Use a weighted loss function during training to reduce the prediction error of minority stress types.

The objective is to enhance the model's generalization ability and practical application value by introducing diverse plant species and stress conditions. These will facilitate the model's effective adaptation to cross-species and cross-stress conditions, enhancing its robustness.

| 1. MFDm^6^ARice for rice m^6^A sites prediction. |
| --- |
| **Input:** Rice m^6^A sequences: $S$, numerical representation: $match\_dict$={A=1, T=2, G=3, C=4, P (pad)=0} |
| **Output:** Probability of a sample containing a m^6^A site: $\hat{y}$ |
| 1: **function** $fea\_encoding(S)$ |
| 2: $F\leftarrow[]$ # [] means list |
| 3: **for** $k\leftarrow1$ **to** $length(S)$ **do** |
| 4: $F_{k}\leftarrow[]$ |
| 5: $F_{k}\leftarrow match\_dict(S_{k})$ |
| 6: $F=F+F_{k}$ |
| 7: **end for** |
| 8: **return** $F$ |
| 9: **end function** |
| 10: **function** $MKFF(F)$ |
| 11: $F_{MKFF}\leftarrow[]$ |
| 12: **for** $i\leftarrow1$ **to** $length(S)$ **do** |
| 13: $F_{MKFF}^{i}\leftarrow[]$ |
| 14: $MK_{i}\leftarrow MK(F_{i}))$ |
| 15: $F_{MKFF}^{i}\leftarrow GLDF(MK_{i})$ |
| 16: $F_{MKFF}= F_{MKFF}+ F_{MKFF}^{i}$ |
| 17: **end for** |
| 18: **return** $F_{MKFF}$ |
| 19: **end function** |
| 20: **function** $DRFE(F_{MKFF})$ |
| 21: $F_{DRFE}\leftarrow[]$ |
| 22: **for** $j\leftarrow1$ **to** $length(S)$ **do** |
| 23: $F_{DRFE}^{j}\leftarrow[]$ |
| 24: $F_{down}^{j}= {MaxPool(Pad(F}_{DRFE}^{j}))$ |
| 25: $F_{DRFE}^{j}=Conv(Pad(Conv(Pad(F_{down}^{j})))) +F_{down}^{j}$ |
| 26: optimization about $F_{DRFE}^{j}$ by contrastive learning loss function |
| 27: $F_{DRFE}= F_{DRFE}+ F_{DRFE}^{j}$ |
| 28: **end for** |
| 29: **return** $F_{DRFE}$ |
| 30: **end function** |
| 31: **function** $Output(F_{DRFE})$ |
| 32: $\hat{y}\leftarrow[]$ |
| 33: **for** $l\leftarrow1$ **to** $length(S)$ **do** |
| 34: $\hat{y}_{l}\leftarrow[]$ |
| 35: $\hat{y}_{l}\leftarrow MLP(F_{DRFE}^{l})$ |
| 36: optimization about $\hat{y}_{l}$ by binary cross-entropy loss function |
| 37: $\hat{y}= \hat{y}+ \hat{y}_{l}$ |
| 38: **end for** |
| 39: **return** $\hat{y}$ |
| 40: **end function** |


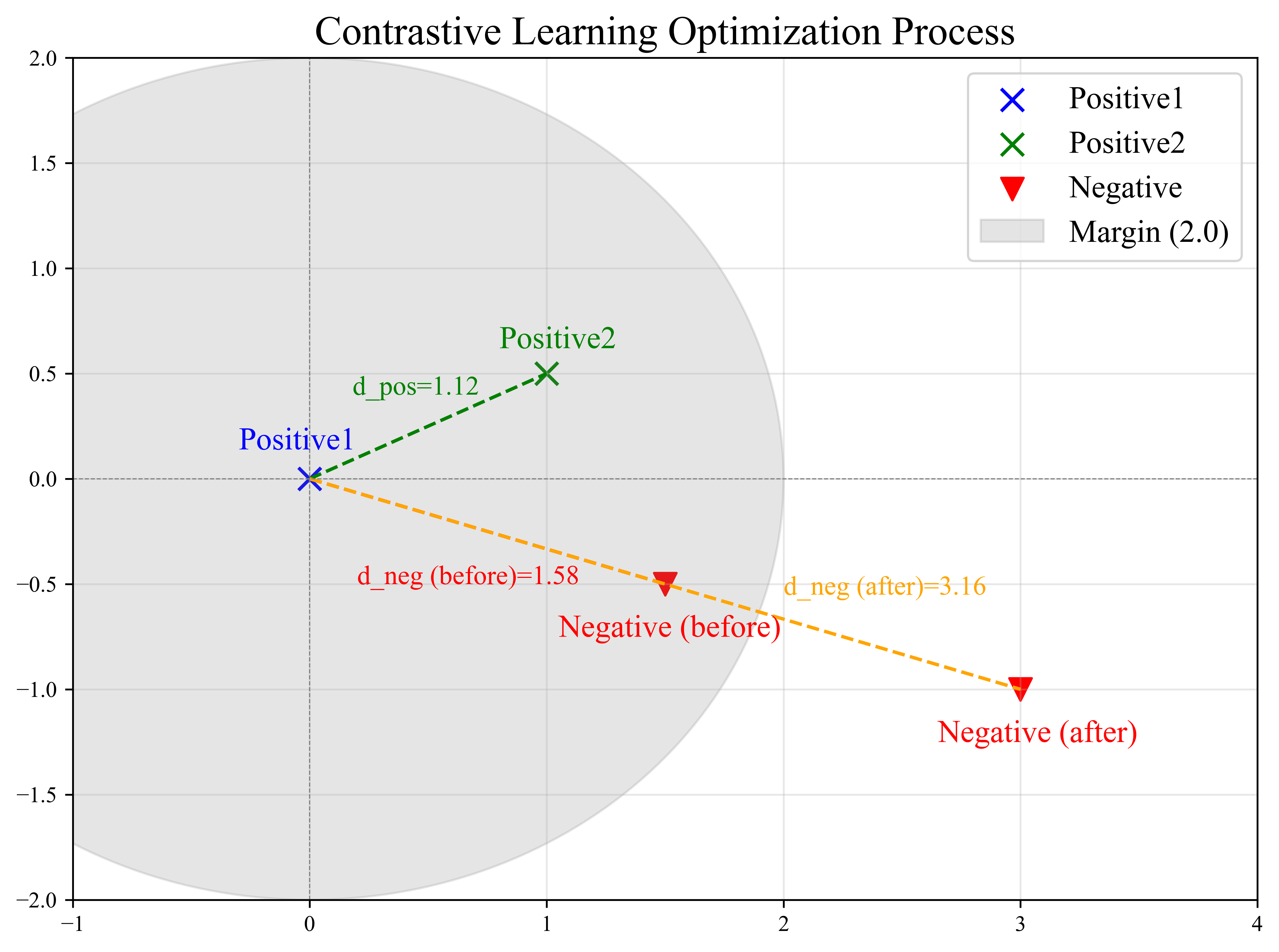


1. Diagram of contrastive learning optimization process.

Positive1 and Positive2 belong to the same class, and their direct distance d_pos is within the margin range, so no optimization is needed. Positive1 and Negative belong to different classes. In the beginning, the distance between them d_neg (before) is less than the margin, so optimization is needed to separate them. After optimization, the distance between them is d_neg (after) = 3.16.


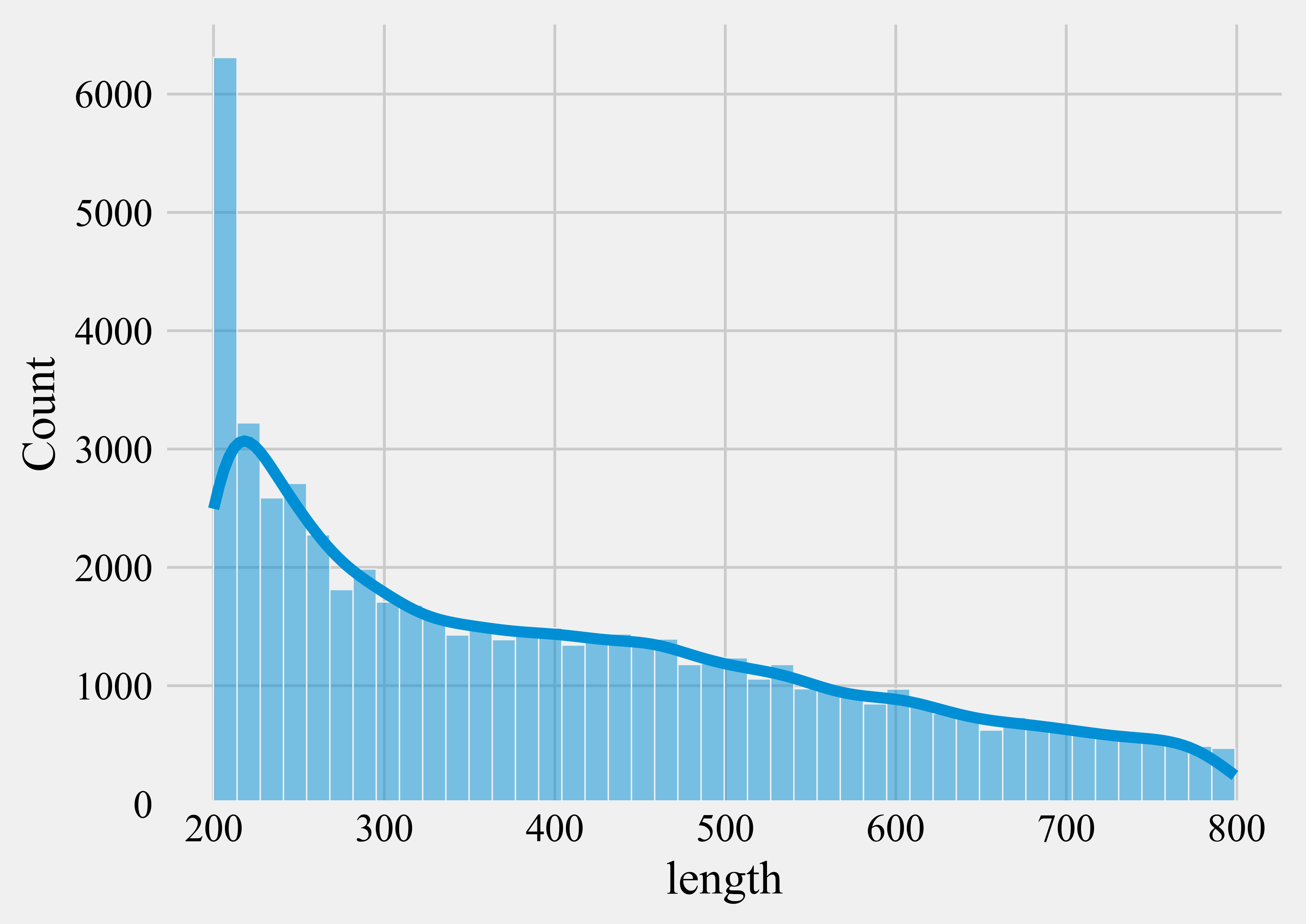


1. Sequence length distribution.


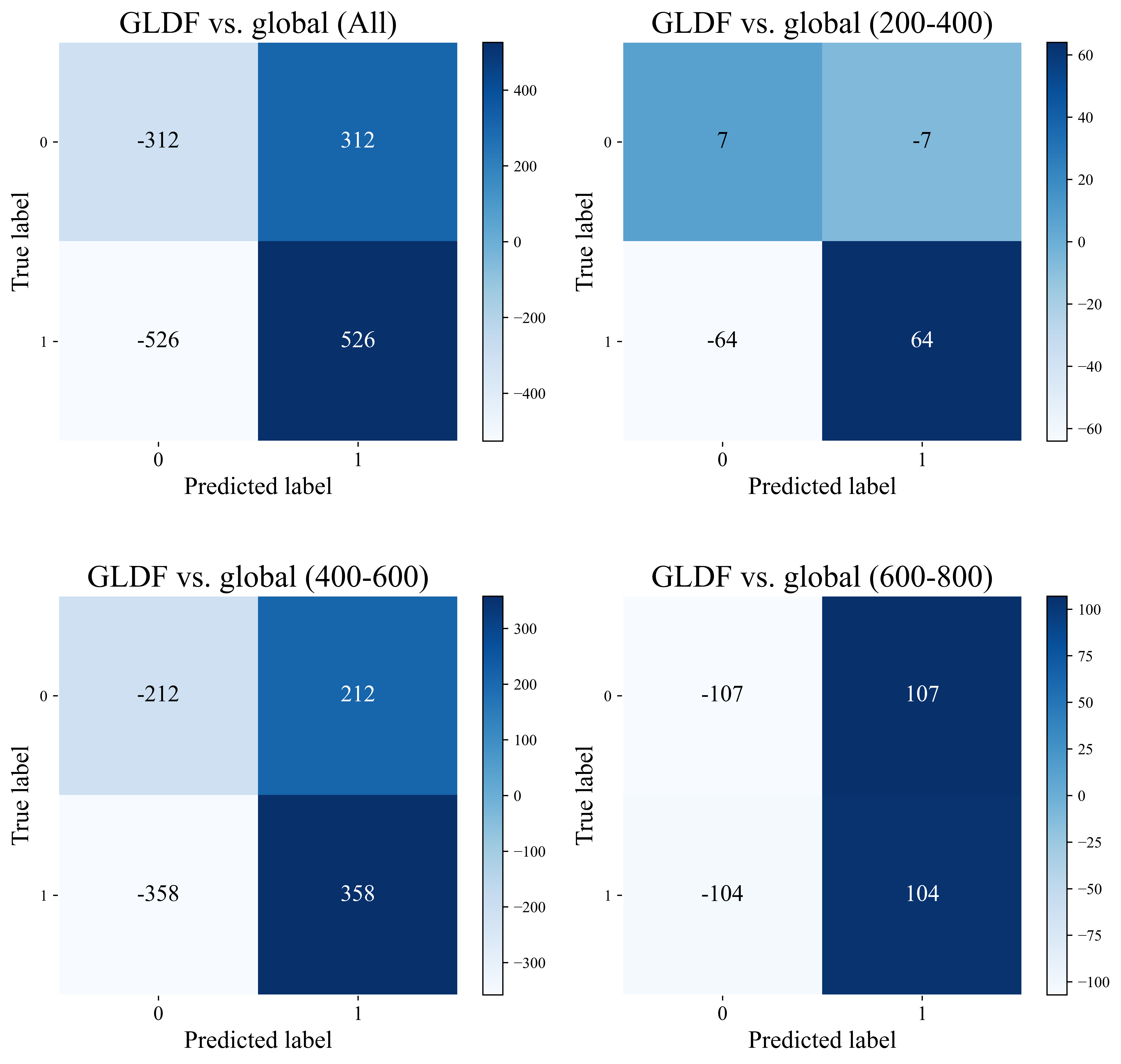


1. The difference between the confusion matrices of GLDF and global variants under different sequence length distributions.


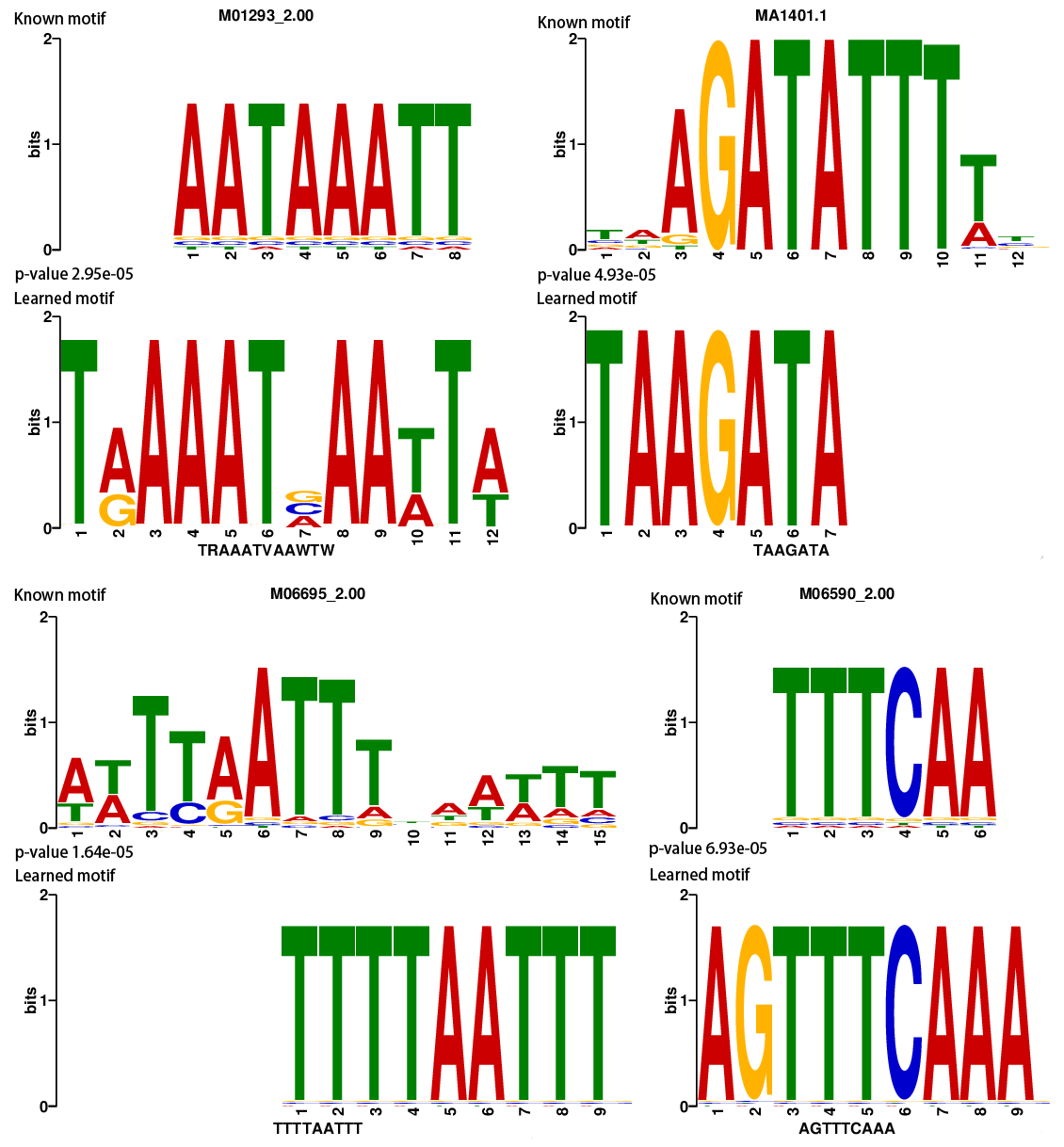


1. Visualization of some significant motifs learned by MFDm^6^ARice.

**Reference**

1. Szegedy C, Ioffe S, Vanhoucke V, et al. Inception-v4, inception-resnet and the impact of residual connections on learning. In: Proceedings of the AAAI conference on artificial intelligence, volume 31. 2017.
2. Bergstra J, Komer B, Eliasmith C, et al. Hyperopt: a python library for model selection and hyperparameter optimization. Computational Science & Discovery 2015; 8(1):01400.
3. Devlin J. Bert: Pre-training of deep bidirectional transformers for language understanding. arXiv preprint arXiv:181004805 2018.
4. Ji Y, Zhou Z, Liu H, et al. DNABERT: pre-trained Bidirectional Encoder Representations from Transformers model for DNA-language in genome. Bioinformatics 2021; 37(15):2112–2120.
5. Church KW. Word2Vec. Natural Language Engineering 2017; 23(1):155–162.
6. DeVito Z. Torchscript: Optimized execution of pytorch programs. Retrieved January 2022.
7. Tang Y, Chen K, Song B, et al. m6A-Atlas: a comprehensive knowledgebase for unraveling the N6-methyladenosine (m6A) epitranscriptome. Nucleic acids research 2021; 49(D1):D134–D143.
8. Barrett T, Suzek TO, Troup DB, et al. NCBI GEO: mining millions of expression profiles—database and tools. Nucleic acids research 2005; 33(suppl 1):D562–D566.
9. Chawla NV, Bowyer KW, Hall LO, et al. SMOTE: synthetic minority over-sampling technique. Journal of artificial intelligence research 2002; 16:321–357.
10. Goodfellow I, Pouget-Abadie J, Mirza M, et al. Generative adversarial networks. Communications of the ACM 2020; 63(11):139–144.
